# Hiatal Hernia Repair with Gore Bio-A Tissue Reinforcement: Our Experience

**DOI:** 10.1155/2014/851278

**Published:** 2014-04-22

**Authors:** Agrusa Antonino, Romano Giorgio, Frazzetta Giuseppe, De Vita Giovanni, Di Giovanni Silvia, Chianetta Daniela, Di Buono Giuseppe, Sorce Vincenzo, Gulotta Gaspare

**Affiliations:** Dipartimento di Chirurgia Generale d'Urgenza e dei Trapianti d'Organo., U.O.C Chirurgia Generale e d'Urgenza, Azienda Ospedaliera Policlinico Universitario “Paolo Giaccone”, Via Liborio Giuffrè 28, Palermo, 90100 Sicily, Italy

## Abstract

Type I hiatal hernia is associated with gastroesophageal reflux disease (GERD) in 50–90% of cases. Several trials strongly support surgery as an effective alternative to medical therapy. Today, laparoscopic fundoplication is considered as the procedure of choice. However, primary laparoscopic hiatal hernia repair is associated with upto 42% recurrence rate. Mesh reinforcement of the crural closure decreases the recurrence but can lead to complications, above all nonabsorbable ones. We experiment a new totally absorbable mesh by Gore. *Case*. We present a case of a 65-year-old female patient with a 6-year classic history of GERD. Endoscopy revealed a large hiatal hernia and esophagitis. pH study was positive for acid reflux; esophageal manometry revealed LES intrathoracic dislocation. With laparoscopic approach, the hiatal hernia defect was identified and primarily repaired, by crural closure. Gore Bio-A Tissue Reinforcement was trimmed to fit the defect accommodating the esophagus. Nissen fundoplication was performed. *Result*. Bio-A mesh was easily placed laparoscopically. It has good handling and could be cut and tailored intraoperatively for optimal adaptation. There were no short-term complications. *Conclusion*. Crural closure reinforcement can be done readily with this new totally absorbable mesh replaced by soft tissue over six months. However, further data and studies are needed to evaluate long-term outcomes.

## 1. Introduction

Hiatal hernia is defined as the transitory or stable dislocation of a part of the stomach in mediastinum through the diaphragmatic crura delimiting esophageal hiatus. Its developing presupposes anatomic anomalies or weakening of structures and mechanisms which are able to maintain esophagogastric junction and stomach in the abdominal cavity [1]. It is a very common pathological condition with a frequency of about 40–60% in adult age [2]. Several studies showed the multifactorial etiology; the congenital ones are due to anomalies developmental abnormalities of the esophagus, like brachyesophagus; certain predisposing factors are obesity, pregnancy, BPCO, and stipsis which are all conditions that increase endoabdominal pressure promoting stomach's migration in the lower thoracic space. In all, these conditions will determinate a positive pressure gradient from peritoneum to pleura that acts on the esophageal hiatus where the esophageal wall is not perfectly adherent to the diaphragmatic muscle but only loosely connected by Bertelli's phrenoesophageal membrane and by loose connective tissue which often are functionally altered [3]. Classically, hiatal hernia was classified in four types using Hill classification [2, 4]; type I (in which esophagogastric junction and cardia are dislocated in the lower mediastinum) is the most frequent, while types II (in which esophagogastric junction and cardia remain in abdomen and are a part of fund of the stomach to migrate in the chest alongside the cardia: “hernia rolling”), III, and IV (in which, due to the progressive enlargement of the anatomical defect, both cardia and other organs can migrate in the mediastinum) are very rare conditions [5]. Type I hiatal hernia is associated with GERD in 50–90% of cases, even if not all patients with hiatal hernias have symptomatic reflux; in fact, its presence gradually compromise esophagogastric junction continence promoting the backwater of acid secretion and its reflux in contact with esophageal mucosa during transient relaxations of the LES and also reducing clearing systems overall for large hiatal hernias [6, 7]. Clinical presentation includes typical symptoms like heartburn and regurgitation and atypical manifestations like laryngitis, asthma, dental erosion, cardiac arrhythmias pharyngitis, sinusitis, and recurrent otitis. The indication for surgery for gastroesophageal reflux has changed in the last 20 years, including unwillingness of patient to take medication for long time, heartburn, and regurgitations noncompletely controlled by mediations; respiratory symptoms induced by GERD; poor patience's compliance with medication; adverse effects of long-term therapy; complications of GERD (Barrett's esophagus and peptic strictures) [8–10]. Several randomized controlled trials with follow-up of studies ranging from 1 to 10.6 years have compared surgical therapy with medical therapy for the treatment of GERD and strongly support surgery as an effective alternative to medical therapy [11–13]. Fundoplication has also been demonstrated to lead to improved or at least comparable quality of life to that of the medically treated patients and is associated with high patients' satisfactions rates. A laparoscopic total fundoplication is considered today as the procedure of choice, because it increases the resting pressure and length of the lower esophageal sphincter decreases the number of transient LES relaxations and improves quality of esophageal peristalsis. This procedure is associated with low morbidity, a short hospital stay, and excellent outcomes; follow-up demonstrates complete symptoms control in 80–90% of patients 10 years after fundoplication. Primary laparoscopic hiatal hernia repair is associated with up to 42% recurrence rate [10, 12]. The literature demonstrates that an incidence of hernia recurrence following hiatoplasty is not negligible. Several technical details such as complete removal of hernia sac, performance of a total fundoplication, fixing the stomach to the abdominal wall, or diaphragmatic crura are recommended by several surgeons to achieve better results but without scientific evidence. This has led to the use of mesh for crural repair, which has resulted in an improved recurrence rate (0–24%) [12, 13]. The idea of applying a mesh to reinforce hiatal closure follows the principle of applying these materials in ventral and inguinal hernia, where it is known to reduce recurrence rates. Several randomized and nonrandomized series of studies were published to understand whether prosthesis use decreases recurrence and whether it is safe in short- and long-term, to definite the ideal prosthetic material, and to clarify whether its shape and type of fixation are used routinely or in special situations [14, 15]. However mesh complications have been observed such as postoperative dysphagia, esophageal mesh erosion, mesh erosion in the proximal stomach, mesh-related paraesophageal fibrosis, stricture and fibrotic encasement of the distal esophagus, and late esophageal perforation after ischemia [16]. All these complications seem to be correlated with the prosthetic material used and with the way in which it is fixed. Many materials are being used and there is no consensus about which is the best; the characteristic of an ideal prosthesis should be as follows: rapid tissue integration, minimal shrinkage, lack of adherence to hollow viscera, and secure attachment. The most common, low cost, and easy handling prosthetic material is polypropylene. In our experience, we prefer to use, according to the last literature indications, a completely absorbable mesh: Gore Bio-A Tissue Reinforcement [17].

## 2. Clinical Case

**Figure 1:**
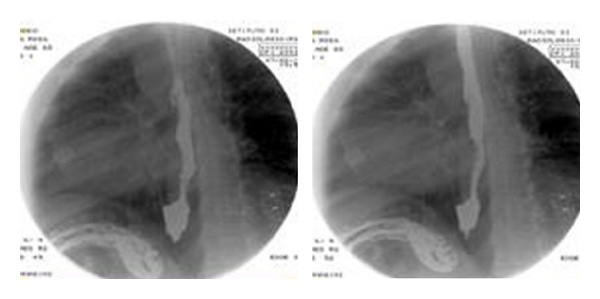
Videofluorographic study showing hiatal hernia and gastroesophageal reflux.

A 65-year-old female patient with a 6-year history of GERD, well controlled by medications, and unwilling to follow long-term therapy came to our attention having heartburn, regurgitation, belching, and dysphonic symptoms. An EGDS was performed showing large type I hiatal hernia > 3 cm with esophagitis signs and island of Barrett's disease to the biopsy. A dynamic Videofluorographic study was also performed, which demonstrates the presence of hiatal hernia and the reflux and backwater of gastric contents in the lower esophagus (Figure 1). To complete the preoperative work-out, the patient underwent a 24-hour pH study, which was positive for acid reflux, and then esophageal manometry which was positive for LES altered function [18–21]. The patient underwent a surgery with laparoscopic technique.

## 3. Our Technique

**Figure 2:**
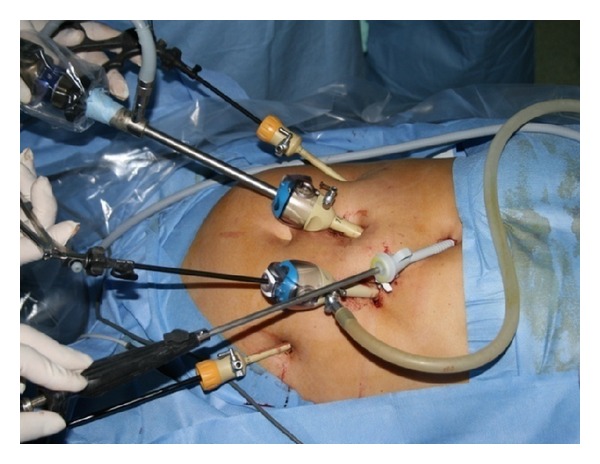
Trocars' placement.

**Figure 3:**
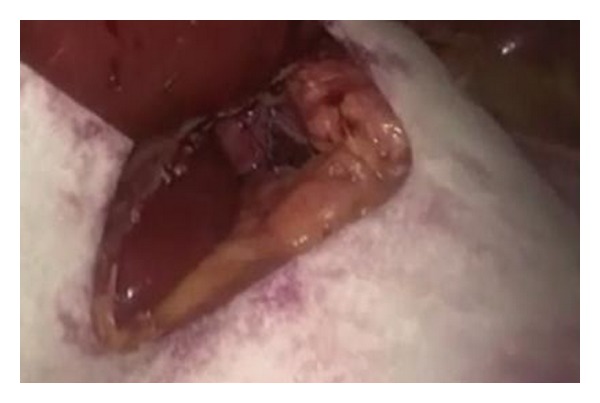
Gore Bio-A Tissue Reinforcement before placing over the crura.

**Figure 4:**
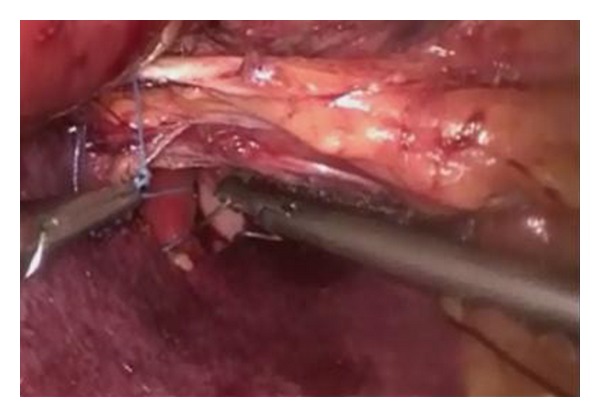
Gore Bio-A Tissue Reinforcement placed over the crura, fixed by absorbable suture.

We led the operation with five trocars, after pneumoperitoneum induction by Veress needle positioned in Palmer's point; we introduced the first 12 mm trocar, for optical system, in mesogastric region 2 cm over the umbilicus. Other two operative 10 mm trocars were placed on the left and the right side 4 cm away from the first; in the end, the last two 5 mm trocars were placed in xiphoid region, for liver divarication, and in pararectal left of subumbilical region for stomach's retraction (Figure 2). After accurate exploration of the all abdominal cavity specially the diaphragmatic abdominal side, to exclude other pathologies, such as diaphragmatic endometriosis [22], we sectioned the phrenoesophageal membrane to expose and reduced hernia's sac; anterior vagus nerve was identified. Then, left and right crura were exposed and a retroesophageal window was created, having care to identify the posterior vagus nerve; webbing was passed under esophagus and it was retracted to the low. After correct and complete diaphragmatic pillars exposition, two nonabsorbable suture size 0 were given to primary closure of the esophageal hiatus [23, 24]. Its function was only to approach the pillars, not for primary lock, trying to evitate any tension. Subsequently Gore Bio-A Tissue Reinforcement absorbable mesh with a “U” shape was positioned to reinforce hiatoplasty (Figure 3). We had care in the correct positioning of the mesh, but nonperfect accommodation of it in the right anatomic region led us to remodeling the mesh so it could better sit over the crura. We paid attention so as not to make contact between the mesh and esophageal wall, positioning it to a 1 cm of distance, to exclude compressive or erosive events to the organ. We put it under posterior esophageal wall over the crura, fixing it with two absorbable 2/0 size suture, to prevent later dislocation or migration (Figure 4). In the end, a Nissen fundoplication was realized with a wrap of 2 cm long (Figure 7). The mesh had its memory but it was handily and very simple to introduce and place. It can be modeled to shape adjustment and better allocation. All the procedure does not enlarge operative time and does not add complication. There was no blood leak. No aspiration drainage was necessary.

## 4. Discussion

**Figure 5:**
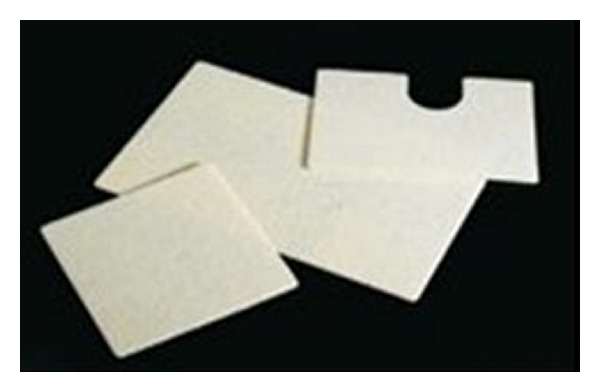
Gore Bio-A Tissue Reinforcement.

**Figure 6:**
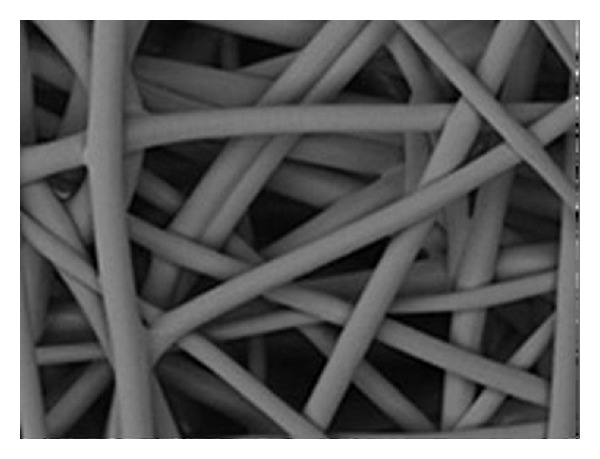
Gore Bio-A Tissue Reinforcement 3D aspect.

**Figure 7:**
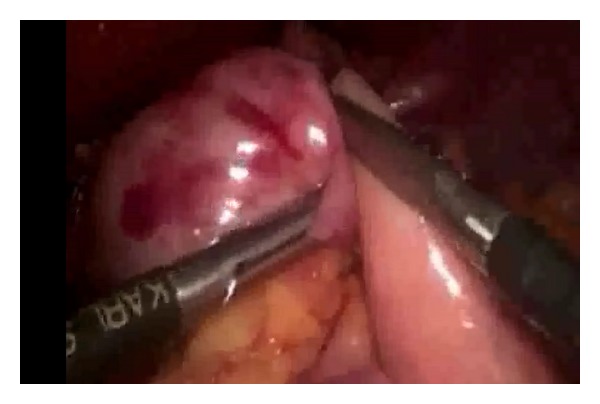
Preparation of gastric fundus for fundoplication.

Gore Bio-A Tissue Reinforcement is a 3D web of completely absorbable synthetic polymers replaced by soft tissue over six months [17, 24] (Figure 5); It is a mix of glycolic acid and trimethylene carbonate and its function is, rather than making a mechanical barrier, stimulating collagens deposition and ingrowths of new connective soft tissue (Figure 6). It was demonstrated that Gore Bio-A increase cellular in-growth in 7–30 days more and more previously than biologics mesh; it also increase new blood vessels formation in 7–14 days reaching the greatest vascular in growth. Instead, the biologic meshes of Gore Bio-A seem to induce the least inflammatory infiltrate. The preformed shape is useful to hiatal hernia repair and its structure made it handy and easy to allocate or modify to a better positioning. To prevent dislocation it can be fixed with absorbable point to the crura or using biologic glues. Our experience was positive above all, because in the 2-month follow-up the patient seems to be going well; she had no more GERD symptoms, no gas bloat syndrome, or bleaching and most importantly no dysphagia was referred. It is clear that Gore Bio-A Tissue Reinforcement seems to have all the best characteristics to hernia hiatal laparoscopic repair reducing both recurrence rates and postoperative mesh related complications, even if several other cases and studies are necessary.
