# Corrigendum to “Hiatal Hernia Repair with Gore Bio-A Tissue Reinforcement: Our Experience”

**DOI:** 10.1155/2015/589098

**Published:** 2015-12-21

**Authors:** Antonino Agrusa, Giorgio Romano, Giuseppe Frazzetta, Daniela Chianetta, Giovanni De Vita, Giuseppe Di Buono, Vincenzo Sorce, Silvia Di Giovanni, Gaspare Gulotta

**Affiliations:** Dipartimento di Chirurgia Generale d'Urgenza e dei Trapianti d'Organo., U.O.C Chirurgia Generale e d'Urgenza, Azienda Ospedaliera Policlinico Universitario “Paolo Giaccone”, Via Liborio Giuffrè 28, Palermo, 90100 Sicily, Italy

In the case report titled “Hiatal Hernia Repair with Gore Bio-A Tissue Reinforcement: Our Experience,” [1] the correct author list should be “Antonino Agrusa, Giorgio Romano, Giuseppe Frazzetta, Daniela Chianetta, Giovanni De Vita, Giuseppe Di Buono, Vincenzo Sorce, Silvia Di Giovanni, and Gaspare Gulotta.”

The first and last names of the authors were reversed. The correct names in order (first name, last name) have been shown in the front matter of the paper.
